# Fabrication of Cu_2_ZnSnS_4_ Thin Films from Ball-Milled Nanoparticle inks under Various Annealing Temperatures

**DOI:** 10.3390/nano9111615

**Published:** 2019-11-14

**Authors:** Xianfeng Zhang, Engang Fu, Maoxi Zheng, Yuehui Wang

**Affiliations:** 1 Zhongshan Institute, University of Electronic Science and Technology of China, Zhongshan 528402, China; zhmx1984@126.com; 2 PANOTECH. INC. 7-13-6 Ginza, Tyouo-Ku, Toyo 1040061, Japan; 3 State Key Laboratory of Nuclear Physics and Technology, School of Physics, Peking University, Beijing 100871, China; efu@pku.edu.cn; 4 Guangdong Engineering-Technology Research Center of Nano-Photoelectric Functional Films and Devices, Zhongshan 528402, China

**Keywords:** Cu_2_ZnSnS_4_ solar cell, annealing, secondary phase

## Abstract

Cu_2_ZnSnS_4_ (CZTS) has been recognized as a promising thin-film absorber material of chalcopyrite-related solar cells. A two-stage method for fabricating CZTS films using CZTS nanoparticles was developed. Nanocrystal inks fabricated by a ball-milling method was utilized to °C deposit CZTS precursors by spin-coating approach. The CZTS precursors were annealed in the sulfur atmosphere under different annealing temperatures ranging from 550 °C to 650 °C. Influences of annealing temperature on grain growth, composition, crystallinity, and photovoltaic properties of CZTS films were characterized. With the increase of annealing temperature, grain growth was enhanced, while the sulfur atomic ratio fist increased then decreased. The crystallinity of the films was significantly improved after the annealing, and the obvious peak of the secondary phase of ZnS, were observed from the X-ray diffraction results, when the annealing temperature increased to 625 °C. However, the secondary phase was not detected from the surface Raman spectrum. Through comparing the Raman spectrum of different areas of the CZTS film, secondary phases of ZnS and SnS were observed, indicating the decomposition of CZTS films, due to the high temperature. The highest conversion efficiency of 7.5% was obtained when the annealing temperature was 600 °C.

## 1. Introduction

The quaternary semiconductor Cu_2_ZnSnS_4_ (CZTS) using just earth-abundant materials and its counterpart Cu_2_ZnSn(S, Se)_4_ (CZTSSe), in which some of the sulfur atoms are replaced by selenium, are very promising thin film photovoltaic absorber layers of solar cell in the future, due to the ideal optical properties, nontoxicity, and low cost of the constituent elements [1,2,3]. 1.5 eV bandgap of the pure CZTS is nearly ideal for single-junction solar cell, and the bandgap can be tuned to less than 1.0 eV by substituting the sulfur atoms by selenium atoms [4]. Due to this merit, CZTS-based devices are expected to have the same theoretical efficiency as Cu(In, Ga)Se_2_ (CIGS) solar cell [5]. Although, up to now efficiency of pure CZTS solar cell has surpassed 10%, while CZTSSe solar cell has achieved a record efficiency of 12.6% by a typical hydrazine-solution based synthesis method recently [6]. However, utilization of hydrazine has limited the popularization because of the toxicity and residual of organic solvents in the film [7]. As a result, alternative solution process route using just non-toxic solvents have been reported by several groups [8,9,10].

In this work, we report our unique approach for fabricating CZTS films by a so-called ‘two-stage’ method from the CZTS nanocrystal inks, that is, CZTS nanoparticles are firstly deposited on the substrate by a spin-coating method to obtain CZTS precursor. The as-deposited precursors are then annealed in the sulfur atmosphere at a high temperature to get large grain size and better crystallinity. Specific details have been revealed in our previous publications [11,12]. It has been reported that the following reaction could move forward or reverse, depending on the annealing temperature and sulfur vapor pressure [13,14]:

(1) $${\mathrm{Cu}}_{2}{\mathrm{ZnSnS}}_{4}⇌{\mathrm{Cu}}_{2}S\left(s\right)+\mathrm{ZnS}\left(s\right)+\mathrm{SnS}\left(s\right)+\frac{1}{2}S_{2}\left(g\right),$$

(2) SnS(s)⇌SnS(g).

In our case, sulfur vapor pressure is also controlled by the annealing temperature. As a result, annealing temperature plays a very important role in the two-stage process. In this paper, our aim is to study the influence of annealing temperature on the properties of CZTS films. Dependence of the morphology, composition, crystallinity, and electrical properties on annealing temperature is investigated.

## 2. Experimental Method

CZTS source powder with 99.999% purity was obtained from High Purity Materials Kojundo Chemical Laboratory Co., Ltd. (Saitama, Japan). CZTS nanocrystal inks were fabricated by ball-milling method, which have been discussed in detail, in our previous work [11]. The CZTS precursor was then deposited on the glass substrate using the as-fabricated inks by a spin-coating approach. The precursor and elemental sulfur powder (99.999%) were placed in an ampoule, which was evacuated to around 2.0 × 10^−3^ Pa, and N_2_ gas (99.99%) was used to fill the ampoule to get a final pressure of around 2.0 × 10^−4^ Pa. Then the ampoule was sealed up to isolate the substrate and sulfur with the surrounding. The annealing was conducted in a cubic annealing furnace with a temperature deviation from the setting value of less than 1% in the atmosphere. N_2_ gas was used as the carrier of heat to enhance heat exchange between the substrate and the furnace and to adjust the partial pressure of sulfur. The total pressure in the ampoule was adjusted to less than 1.0 × 10^−5^ Pa atm during the annealing process by presetting the pressure of N_2_. The annealing temperature was selected between 550 °C and 650 °C with an interval of 25 °C for each sample, and, in total, five temperatures were conducted. The annealing furnace was set at a heating rate from room temperature to the target temperature of 15 °C min^−1^. The total annealing time was chosen to be 3 h. After the annealing was finished, the ampoule was cold together with the furnace naturally to around 400 °C to provide the substrate with sulfur atmosphere protection, since lack of sulfur vapor will lead to decomposition of CZTS films at a high temperature, of over 400 °C, judging from reaction (1). Then the ampoule was withdrawn to the atmosphere, cooling down to less than 200 °C within 5 min to get rid of sulfur condensation on the surface of CZTS film. Comparing with Reference [11], experimental parameters in this work were greatly different. Firstly, in Reference [11], it was a rapid annealing process, during which the increasing rate of annealing temperature was 40 °C min^−1^, while in this work, it was 15 °C min^−1^. Rapid temperature increase could generate temperature nonuniformity, leading to ununiform annealing result. Secondly, the annealing process at 600 °C lasted for 20 min in Reference [11]. In this work, it was 3 h. The raised annealing temperature can promote crystallization and grain growth of CZTS films. Finally, in this work, the ampoule was filled with N_2_, which promoted heat transmission and ensured the sample temperature is the same with setting temperature. In Reference [11], the ampoule was set with high vacuum, and heat transmission was dominated by radiation. Together with the short annealing time, it makes temperature of the substrate deviated from the setting value. Thus, the annealing result could be different for the two works.

Then, the as-grown CZTS film was taken out of the ampoule and used to conduct characterization immediately. To check photovoltaic properties of CZTS films, the full solar cell structure was completed, as follows: A 50-nm-thick CdS buffer layer was first deposited on CZTS film by chemical bath deposition; then layers of metal-organic chemical vapor deposited i-ZnO (80 nm) and B-doped ZnO (600 nm). Finally, a front-contact Al grid was deposited on the top via evaporation method.

The morphology of the CZTS films was characterized with a scanning electron microscope (SEM) equipped with a JED-2300T energy dispersive spectroscopy (EDS) system (JSM-7001F, JEOL, Tokyo, Japan) operating at an acceleration voltage of 10 kV. EDS was measured at an acceleration voltage of 15 kV for compositional analysis. Crystallization of CZTS film was performed by X-ray diffraction (XRD) (SmartLab, Rigaku Corporation, Tokyo, Japan) with a voltage of 40 kV and 20 mA current. Raman spectra was measured by a Raman spectrometer (RS-1000, JASCO Corporation, Tokyo, Japan) with a resolution of 1.6 cm^−1^. Solar cell performance was measured with a 913 CV type current-voltage (J-V) tester (AM1.5) provided by a solar simulator (LP-50B, EKO, Tokyo, Japan). The simulator was calibrated by a standard GaAs solar cell to get the standard illumination density (100 mW/cm^−2^). Quantum efficiency (QE) of CZTS solar cell was characterized by a QE tester (QE-2000, Otsuka Electronics Co., Ltd., Osaka, Japan).

## 3. Results and Discussions

### 3.1. Surface and Cross-Sectional SEM

**Figure 1:**
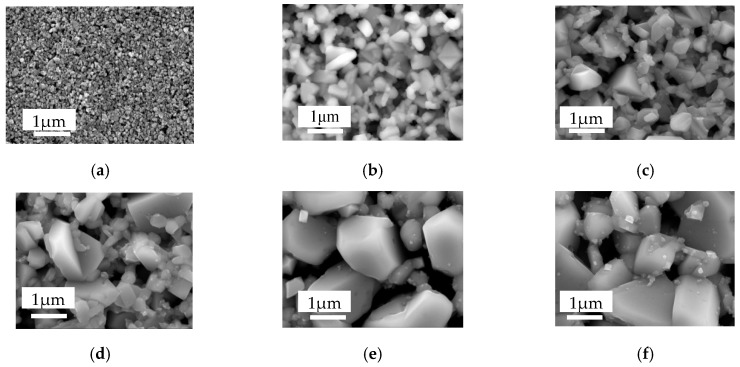
Surface morphology of CZTS films; (**a**) precursor; (**b**) 550 °C; (**c**) 575 °C; (**d**) 600 °C; (**e**) 625 °C; (**f**) 650 °C.

**Figure 2:**
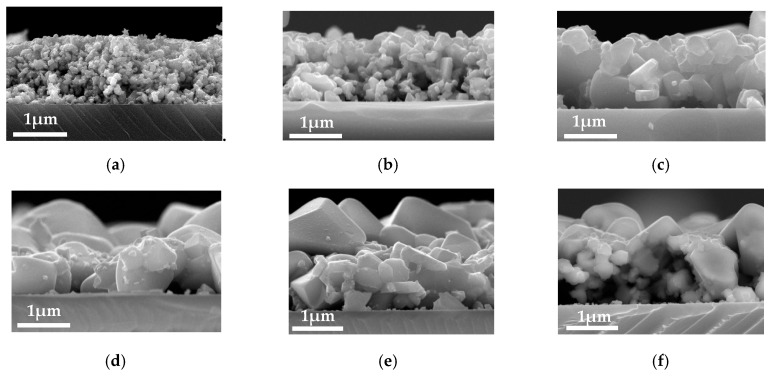
Cross-sectional morphology of CZTS films; (**a**) precursor (**b**) 550 °C; (**c**) 575 °C; (**d**) 600 °C; (**e**) 625 °C; (**f**) 650 °C.

Surface SEM micrographs of the CZTS precursor and some of the annealed films are shown in Figure 1. The precursor shows a very small grain size of several-ten nm, as shown in Figure 1a. After the precursor being annealed under a temperature of 550 °C (Figure 1b) the grain size significantly increases. The whole film morphology can be described as large grains up to 1 μm embed in the small grains of several-hundred nm. With increasing annealing temperature from 550–650 °C the grain size increased gradually. The basic morphology for annealing temperature 550–600 °C (Figure 1b–d) are identical; however, when the temperature is raised to 625 °C the large grains over 1 μm begin to dominate, accompanied by a small number of small size grains (Figure 1e). Grains continue to increase over 2 μm as the temperature is increased to 650 °C (Figure 1f). Cross-sectional SEM micrographs of the annealed films with different annealing temperatures are shown in Figure 2. Cross-sectional image for precursor is absent because it is difficult to prepare appropriate SEM sample, due to the low combining power between the particles. (When one breaks off the substrate, the fresh cross-section is too rough to be observed by SEM). As can be seen from the figures, with the increase of annealing temperatures, the grain size of CZTS films increases significantly, especially when the temperature is over 625 °C large grain of over 2 μm dominates, which is consistent with the surface SEM results. However, pinholes stretching throughout the film in the thickness direction begin to show up, meaning deterioration of the film quality, due to the film decomposition.

### 3.2. Composition of CZTS Films

Table 1 shows composition of CZTS films annealed with different temperature. The precursor shows a sulfur and zinc poor composition comparing to the high quality CZTS films reported by other groups [15,16]. After the film is annealed at a nominal temperature of 550 °C in our case, the composition becomes sulfur-rich, and when the annealing temperature is increased to 575 °C, the sulfur ratio continues to increase. However, when the temperature is further increased, the sulfur ratio begins to decrease and get the minimum value of less than 50%. This result can be explained, as follows: There is a balance between the absorption and emission of sulfur during the annealing process related to the sulfur pressure and substrate temperature. When the annealing temperature increases, sulfur vapor pressure increases as well, which promote the absorption of elemental sulfur. However, when the substrate temperature goes over 600 °C, the emission of sulfur is significantly enhanced; leading to sulfur loss and sulfur-poor composition. Meanwhile, the Sn ratio varies a little even the temperature ranges up to 650 °C, indicating low loss of tin.

### 3.3. X-ray Diffraction and Raman Scattering Spectra Characterization

**Figure 3:**
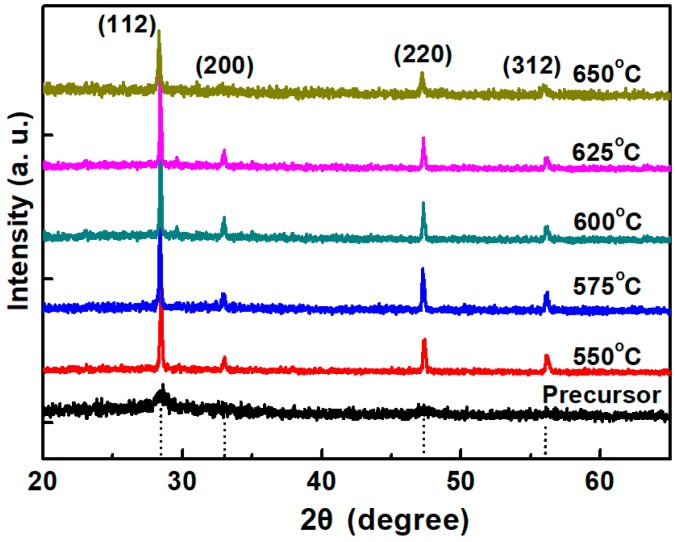
XRD pattern of CZTS precursor and annealed films.

**Figure 4:**
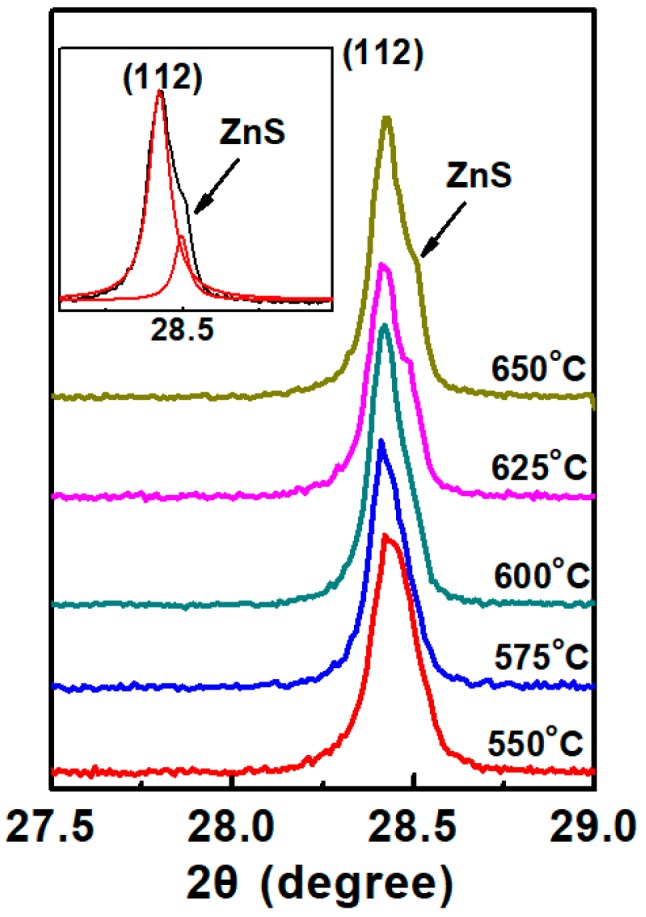
XRD pattern of (112) plane of annealed CZTS films.

XRD patterns of CZTS films are shown in Figure 3. The phase identification pointed to the presence of kesterite structural CZTS films according to Reference [17]. The precursor shows very weak diffraction peaks and just one broad peak around 2θ = 28.5° can be observed, indicating low crystallinity. The 550 °C annealed CZTS film exhibits four peaks at 2θ = 28.5°, 33.0°, 47.3° and 56.5°, corresponding to the peaks of (112), (200), (220) and (321) of kesterite phase CZTS structure, respectively. When the annealing temperature increases, the intensity of the diffraction peaks also increases and gets the maximum value when the annealing temperature is 600 °C, meaning the improvement of the crystallinity. For 625 °C and 650 °C annealed films, the peak intensity slightly decreased, which can be explained by Figure 4 that shows the XRD pattern of (112) plane of annealed CZTS films with different annealing temperatures. For 550 and 575 °C annealed films, the XRD pattern shows a single (112) peak, illustrating no secondary phases can be detected. However, when the annealing temperature is increased to 625 °C, a slight hump begins to show up at the right side of the peak of (112) plane, which is attributed to the peak of ZnS [18], indicating secondary phase. Judging from reaction (1), as has been mentioned above; when the annealing temperature is over 600 °C, the reaction will go the forward direction. The formation of ZnS will generate a correspondent peak in the XRD pattern.

**Figure 5:**
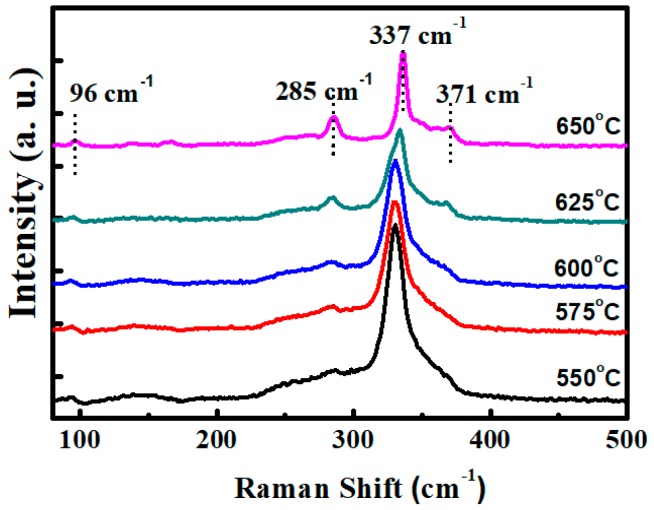
Raman spectra of CZTS films with different annealing temperatures (magnification of object lens: 100×).

**Figure 6:**
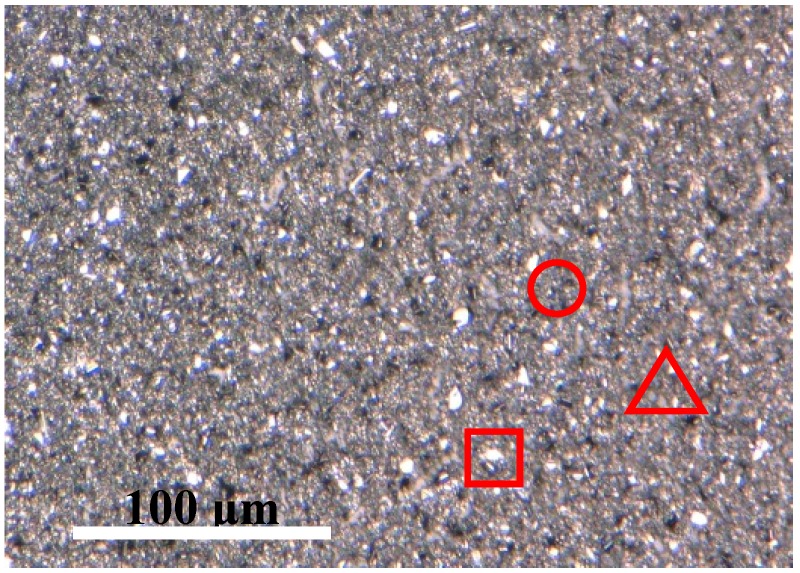
3D microscope image of CZTS film annealed at 625 °C. The marks illustrated the area where Raman measurement were conducted.

**Figure 7:**
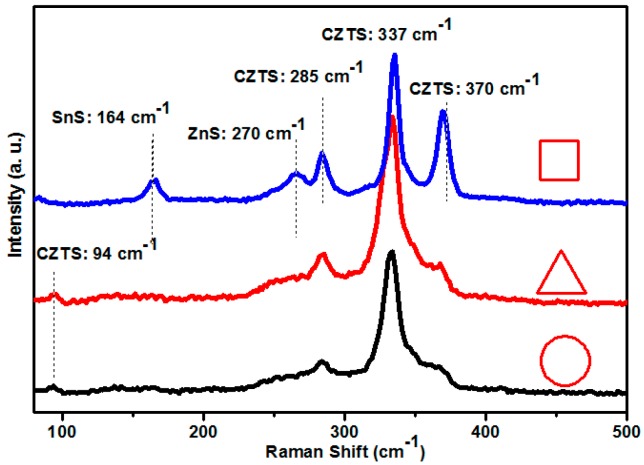
Raman spectra of different areas marked in Figure 5 (magnification of object lens: 0.9×).

To further clarify the secondary phase, surface Raman scattering measurement was conducted with 532 nm excitation wavelength. Figure 5 shows the Raman scattering spectra of CZTS films with different annealing temperatures. The peaks are marked with correspondent phases. Judging from the Raman result, all the phases can be assigned to kesterite CZTS film. The films exhibit a typical Raman spectrum of kesterite CZTS films. The two main peaks of 285.4 and 337 cm^−1^ are corresponding to two A modes, and the shoulder at 372 and a minor peak at 96 cm^−1^ are corresponding to two of the B symmetry modes of the CZTS kesterite structure [19,20,21], respectively. Even though obvious ZnS peak is observed in the XRD spectra, no secondary phases have been detected by the Raman spectrum, although detect of secondary phases for Raman is easier than XRD. A possible explanation is that distribution of secondary phases was ununiform, and the measured area has a small amount. Meanwhile, the spectral resolution was insufficient to distinguish the ZnS peak, which was superimposed by the peaks of CZTS. Figure 6 is a 3D microscope image that illustrates the different areas where Raman measurement has been performed: The circle area is for the normal flat area, the triangle represents flat area mixed with larger particles, and square shows the large particle area. In order to further identify the reason for these results, Raman measurement is conducted on a different area of a CZTS film annealed at 625 °C, as shown in Figure 7. According to the Raman spectrums, only classic peaks of kesterite CZTS film can be observed in the normal flat area. For the marked triangle area, a shoulder of CZTS peak (285 cm^−1^) appears near 270 cm^−1^. It is inferred that it belongs to the A_1_ mode of ZnS [22,23], which is consistent with our XRD result. For the square marked area, besides the peak of 270 cm^−1^, a minor peak at 164 cm^−1^ is present, corresponding to the SnS compound [24,25]. The presence of the peaks of ZnS and SnS illustrate the decomposition of CZTS during the high temperature process. However, Cu_3_SnS_4_ reported somewhere else [26] was not detected in our samples.

**Figure 8:**
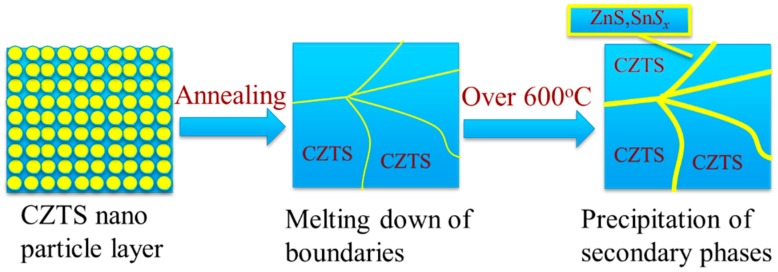
Growth mechanism of CZTS films.

The schematic of the growth mechanism for CZTS films was shown in Figure 8. During the annealing process, with the increase of temperature grain boundaries gradually meltdown, and grain size became larger. Thus, annealing process played a key role in grain growth. However, when the annealing temperature was over 600 °C CZTS crystal started to decompose at the grain boundary, leading to the formation of secondary phases at the boundary and extremely large grains formed, which caused surficial nonuniformity, as shown in Figure 2e,f. Thus, secondary phases, such as ZnS and SnSx were detected when the annealing temperature was 625 °C.

**Figure 9:**
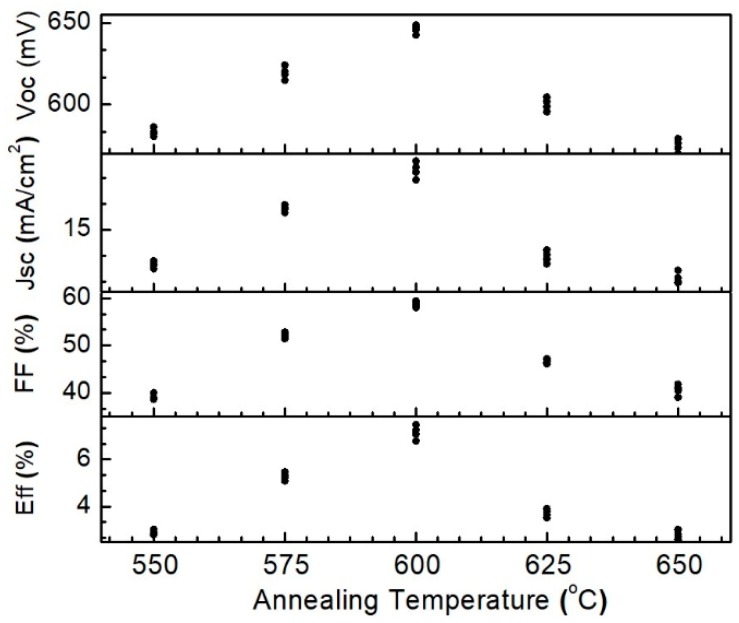
Dependence of solar cell performance on the annealing temperature.

The CZTS films were then fabricated to full solar cell structure, and solar cell performance was evaluated under standard conditions with irradiation of 100 mW/cm^2^. The intensity of the solar simulator was calibrated by a high-precision monocrystalline Si solar cell to achieve a standard illumination. Figure 9 shows the photovoltaic characteristics of CZTS solar cells annealed at different temperature with an area of 0.2 cm^2^. Considering of system errors and circumstances of experiment, the measurement was carried on four samples for each annealing temperature. When the annealing temperature was 550 °C, the conversion efficiency of CZTS solar cell is about 2.8%, with a low open-circuit voltage (Voc) of about 586 mV, a short circuit current (Jsc) of 12.5 mA/cm^2^, and fill factor (FF) of 38.5%. It was inferred that the low performance of the solar cell was due to the low crystallinity of CZTS film because of low annealing temperature. With the increase of annealing temperature, the crystallinity of the absorber layer was improved, which lead to better solar cell performance. The CZTS solar showed a conversion efficiency of around 5.2% (Voc: 618.5 mV, Jsc: 16.3 mA/cm^2^, FF: 51.9%) and the highest value of 7.5% (Voc: 647 mV, Jsc: 16.3 mA/cm^2^, FF: 51.9%) was obtained when the annealing temperature was 600 °C. However, when the annealing temperature further increased, solar cell performance began to deteriorate, as shown in the figure. One explanation was the formation of secondary phases because of the decomposition of CZTS films.

**Figure 10:**
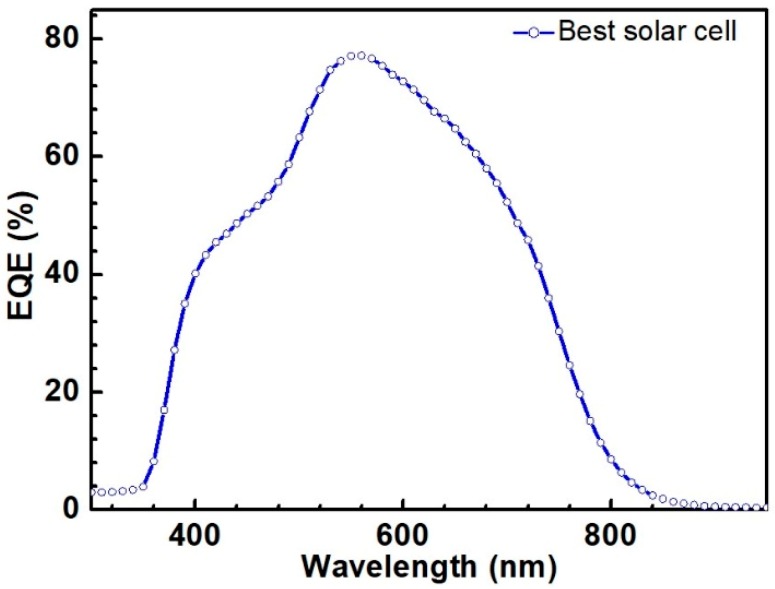
Quantum efficiency of the best CZTS solar cells annealed at 600 °C.

Figure 10. shows the external quantum efficiency (EQE) curve of the best CZTS solar cell with the annealing temperature of 600 °C. The QE curve shows an abrupt drop near the wavelength of 770 nm, which was attributed to CZTS absorption edge. Thus, the bandgap of the CZTS films was calculated to be about 1.61 eV. The features near 510 nm and 380 nm corresponded to the absorption edges of the CdS and ZnO layers [27], respectively, which was common when using the CdS buffer and ZnO window layers. Based on the *EQE* data of a solar cell, *J_SC_* was calculated as [28]:

(3) $$J_{sc}=q\int_{0}^{\infty }QE\left(E\right)b_{s}\left(E,\;T_{s}\right)dE,$$

where *q* is the elementary charge, *QE* is quantum efficiency, and *b_s_* is solar flux or irradiation. The sunlight spectrum data are available from Reference [29] for air mass 1.5. Based on Equation (1), Figure 10 and the solar irradiation spectrum, *J_SC_* of the CZTS solar cell was calculated as 17.5 mA/cm^2^ for a solar cell annealed at 600 °C. It can be seen that *J_sc_* calculated from the *QE* curve deviated from the J-V curve. One explanation for the result is that the J-V curve represents the real performance of a photovoltaic device, but the *QE* measurement was carried out at a single wavelength with much lower intensity than that of a one sun irradiation.

## 4. Conclusions

CZTS precursor is deposited by nanocrystal ink using a spin-coating method. The precursor is then annealed in the sulfur atmosphere under different temperatures. Raising temperature can promote the growth of grain size of CZTS films and when the temperature is over 625 °C pinholes that stretch through the film begin to appear. Increasing temperature can increase the sulfur vapor pressure as well, leading to the conversion of sulfur-poor films to sulfur-rich films. According to the XRD pattern, the crystallinity of CZTS films is significantly improved by the high temperature, and secondary phase of ZnS begins to show up when the annealing temperature is increased to 625 °C. Judging from the Raman scattering spectra, the secondary phase of ZnS mainly exist in the large particles on the surface of the film, related to the decomposition of CZTS films. With the increase of annealing temperature, solar cell performance increases and the best conversion efficiency of 7.5% is obtained when the annealing temperature is 600 °C.

## Figures and Tables

**Table 1:** Composition of CZTS films annealed with different temperature.

Temperature (°C)	Cu	Zn	Sn	S	Zn/Sn	Sn/(Zn + Sn)
Precursor	25.6	9.7	15.1	49.6	0.64	0.61
550	25.0	10.0	14.9	50.1	0.67	0.60
575	24.1	9.6	15.0	51.3	0.64	0.61
600	25.1	9.3	15.4	50.2	0.60	0.62
625	24.7	9.8	15.1	50.5	0.65	0.61
650	25.3	10.6	15.0	49.1	0.70	0.59
